# Predation in Organic and Free-Range Egg Production

**DOI:** 10.3390/ani10020177

**Published:** 2020-01-21

**Authors:** Monique Bestman, Judith Bikker-Ouwejan

**Affiliations:** Louis Bolk Institute, Kosterijland 3-5, 3981 AJ Bunnik, The Netherlands; judith-ouwejan@hotmail.com

**Keywords:** predation, mortality, free-range laying hens, organic laying hens

## Abstract

**Simple Summary:**

On organic and free-range poultry farms, free-range is provided for animal welfare reasons. However, organic/free-range farmers report sightings of birds of prey and foxes attacking their chickens and, as they are regularly finding chicken carcasses in the free-range, they attribute the death of those chickens to predators. In addition, and in contrast to indoor poultry farmers, they report hundreds of missing chickens at the end of the production period. They assume these chickens have left the free-range at the hands of predators. This study investigated whether birds of prey kill chickens on organic/free-range egg production farms, and their impact, in terms of numbers of chickens and yield losses. Field observations were done on 11 organic/free-range farms and, in an online survey, organic/free-range farmers were asked about their farm records. During 79 observations at those 11 farms, a bird of prey was seen 141 times, including 16 attacks by common buzzards and northern goshawks. Based on the results from the online survey (n = 27 farms experiencing predation), on average, 3.7% of the hens in organic/free-range flocks were estimated to have been killed by predators. After calculating missed yield per killed hen, it was roughly estimated that, per flock, predation caused yield losses of EUR 5700 on an average organic farm (size 12,700 hens), and EUR 6700 on an average free-range farm (size 25,000 hens).

**Abstract:**

On organic and free-range poultry farms, a free-range is provided for animal welfare reasons. However, farmers report sightings of birds of prey and sometimes foxes or other predators within the free-range areas. In addition to seeing actual attacks, they also find chicken carcasses in the free-range, the deaths of which they attribute to predators. In addition, and in contrast to indoor poultry farmers, organic/free-range farmers report hundreds of chickens missing, per flock, when comparing the slaughterhouse arrival numbers with farm mortality records. The farmers assume these missing animals are hens that vanished from the free-range area and that predation is the major cause for their disappearance. If so, predation may impact farm yields. This study investigated whether birds of prey kill chickens on organic/free-range egg production farms and the impact, in terms of numbers of chickens and yield losses. This study was to provide qualitative and quantitative information in support of chicken mortality caused by birds of prey. Data were collected through field observations on organic/free-range farms (n = 11) and an online survey among organic/free-range farmers. Seventy-nine field observations on 11 farms resulted in 141 sightings of birds of prey, mostly common buzzards (*Buteo buteo*) and northern goshawks (*Accipiter gentilis*). Forty-four dead hens were found, 36 of them were very likely killed by either birds of prey or foxes. Sixteen attacks on hens by goshawk or buzzard were seen. There were no reasons to assume the attacked hens were in a poor condition prior to the attack. From responses to the online survey (n = 27 farms experiencing predation), it was estimated that on average 3.7% of hens of organic/free-range flocks were killed by predators, while total mortality was 12.2%. After calculating missed yield per killed hen, it was roughly estimated that per flock, predation caused yield losses of EUR 5700 on an average organic farm (size 12,700 hens) and EUR 6700 on an average free-range farm (size 25,000 hens).

## 1. Introduction

In the Netherlands, on 1 January 2019 [1], 6.3 million free-range hens and 2.4 million organic hens were being kept, each of them having 4 m^2^ of free-range area at their disposal, on 252 and 190 farms, respectively.

The poultry are kept in free-range areas for animal welfare reasons. However, predation may cast a shadow on the welfare of chickens and cause economic losses. Predator-related deaths were reported by 40% of Dutch flocks of organic laying hens [2]. A similar situation applies to the free-range poultry in other countries. Predation was estimated to be the cause of death for 0.5% (up to 12%) of laying hens and geese in the United Kingdom [3], 6.3% (up to 34%) of broilers in France [4], 6.7% of laying hens in Switzerland [5] cited in [6], up to 14.2% of laying hens in Denmark [7] and 9.5% (up to 23.5%) of laying hens in Germany [8] cited in [6].

Poultry farmers regard chicken mortality as an economic loss, especially because they believe the predators also, or even mainly, kill healthy, productive hens. Generally speaking, Dutch authorities provide compensation to farmers for the damage caused by protected wildlife (i.e., that cannot be hunted), such as birds of prey, but predation of free-range chickens is not officially recognized as wildlife damage. Prevention of predation is only possible to a limited extent. Predation by foxes can be prevented by fencing in the free-range area and by ensuring that all chickens spend the night inside a fox-proof hen house. In the Netherlands, under certain conditions, hunting licenses are issued to local hunters to kill foxes. Prevention of predation by birds of prey is much more difficult: these birds hunt in the daytime when the hens have access to the free-range. Although netting a range might appear a solution to protect the hens, covering ranges of 5 or 10 hectares (sizes are based on average Dutch organic and free-range farms, respectively [1]) is considered impractical, also because they sometimes contain trees, ditches or large grazing animals. Moreover, farmers applying for municipal environmental permits for such large ‘roofs’ would meet with legal difficulties, and face regulations concerning the aesthetic aspects of large structures within the countryside. Finally, farmers consider such large covers to be too expensive.

The main subject of this study concerned whether birds of prey cause damage on organic/free-range egg production farms and to what extent this damage can be estimated. This study was to provide qualitative and quantitative information in support of chicken mortality caused by birds of prey.

We formulated the following three research questions, in consultation with poultry farmers, people from the wildlife damage commission (BIJ12-Faunafonds) and a birds of prey expert [9]:

1. Which bird of prey species kill hens?
2. Are there any particularities perceptible concerning the condition of hens prior to the attack that may give an impression of their health status?
3. What is the impact of predation, in terms of numbers of hens being killed and the related estimated yield loss?

## 2. Materials and Methods

We addressed these questions by conducting field observations in free-range areas, an online survey among poultry farmers and model calculations. Field observations and video recordings were made to provide qualitative data used to answer questions 1 and 2. The aim of the online survey was to provide quantitative data (e.g., estimations of the numbers of hens killed by predators) for the calculations used in answering question 3. Other sources of such quantitative data consist of key figures and prices published biennially by Wageningen Livestock Research [10].

### 2.1. Field Observations and Video Recordings

Poultry farms with bird-of-prey-related mortality were approached to participate by ‘Pluimveehouderij’, a Dutch magazine for poultry farmers. This resulted in 11 farms, experiencing bird-of-prey-related mortality and that were keeping hens in the period the field observations were planned to take place, namely in July to November 2015.

An observation protocol was created based on a farm visit, together with representatives from the organic poultry farmers union, the wildlife damage commission (BIJ12-Faunafonds) and a bird of prey expert [9]. The observations were done on 11 farms in total. Per observation day, two farms were visited in succession. On the first farm, the free-range was inspected on foot, looking for dead, visibly ill or otherwise impaired hens. The check for the presence of visibly ill or otherwise impaired hens would help to say something about the condition of hens prior to an attack, in case during the following observation an attack would take place. The most commonly observed behaviour in free-range hens includes standing still, pecking, walking and foraging [11]. Hens were considered ‘healthy’ when, in addition to these alternating behaviours, no other peculiarities were seen in behaviour or appearance that would suggest the hens were somehow impaired or diseased. For all carcasses or their remains found, the cause of death was determined on the basis of three categories: fox, bird of prey, or other/unknown. A killing was attributed to a fox when the hen had been decapitated and/or if feathers were gnawed [4,12]. A bird of prey was deemed responsible if parts of the hen had been eaten and feathers were pulled out [4,12]. The third category, ‘other/unknown’, contained any other cause of death. The dead hens were photographed for documentation and evidence. After the inspection of the area on foot, 90-min observations were conducted from under a camouflage net, at a location (inside or outside the free-range) with a clear view either of as much as possible of the free-range area or of a specific spot in the free-range; for example, an area with regular evidence of predation, such as carcasses. On the second farm of the day, the free-range was not entered for biosecurity reasons, in order to prevent diseases from being transmitted. The 90-min observations on this second farm were conducted from outside the free-range; if possible, from a car. After a couple of days, the same farms were visited but in reverse order, in order to observe each free-range at different times of the day. From July to November, as many observations were done as possible; several times on two days close to one another. The final number of observations per farm would depend on the age of the hens and whether there was indeed predation. All observations took place when the pop holes were open; from 8:30 to 20:30, depending on the time of the year. During the 90-min observations, all sightings of birds of prey and their behaviour were noted down in a semi structured way: bird of prey species and behaviour, behaviour of the hen during an attack, and condition of the hen prior to the attack (dead, visibly ill/impaired, healthy), based on the criteria mentioned above. Where possible, photographs were made of birds of prey and their attacks.

On the free-range of one farm, attacks took place repeatedly at the same spot. A wireless surveillance camera (RDI Technology (Shenzhen, China), type CM812732) was installed to make continuous recordings over the course of 16 days. The recordings were in full colour during the day and in black and white between sunset and sunrise. Attacks recorded by this camera were described in the same semi-structured way as described above for the live observations.

### 2.2. Online Survey

The questions were formulated in consultation with representatives of the organic poultry farmers union, three bird of prey experts, a representative from the Dutch Ministry of Economic Affairs, and a communications expert. The questions included ones about production systems (organic/free-range), number of hens, percentage of hens observed on the range under optimum conditions, whether farmers detected mortality caused by predators; and some figures from their last culled flock—number of hens at start, number of hens who died from disease, those found dead on the range who were not killed by predators, those found dead on the range who were killed by predators, the number of hens missing after the count at the slaughterhouse, and the suspected reasons for their absence. Poultry farmers were approached by agricultural magazines ‘Pluimveehouderij’ and ‘Boerderij’ to fill in the online survey about predation, and, in an email, the approximately 50 members of the organic poultry farmers union were asked to do the same. Also, farmers without predation were invited, and we mentioned that we were curious as to why they had no predation. The survey was set up by MWM2 (https://www.mwm2.nl/) and remained available online for 50 days. MWM2 subsequently presented the answers in MS Excel format. The main criterion for including participants’ responses in our analyses was that they answered all quantitative questions about their last culled flock.

### 2.3. Calculation of Yield Losses

To calculate the yield losses due to predation, yields and costs were compared between hens slaughtered at the end of the laying period and those killed by predators exactly halfway through the laying period. Because there was no information about when during the laying period (beginning, middle, end) predation occurred, we assumed the deaths were evenly distributed over the laying period, meaning the same numbers would be killed before the middle of the laying period as after it. Therefore, we calculated ‘hens killed by predators’ to all have been killed halfway through the laying period. Since costs related to young hens and feed differed between organic and free-range farms, the yield losses were calculated for both production systems. Key figures and prices were obtained from the manual ‘Quantitative information animal production 2018–2019′ [10], average farm sizes for organic and free-range farms were used [1], and the percentage of hens assumed to be killed by predators was derived from our own online survey. The calculations were done for brown hens, since these were the only genotypes for which key figures and prices were available. This was the most kept genotype on organic/free-range farms.

## 3. Results

### 3.1. Characteristics of the Farms Included in the Study

From July to November 2015, 79 field observations were conducted on 11 farms. Table 1 shows some of the farm characteristics. 

Table 2 shows date and times of the observations.

### 3.2. Observed Birds of Prey, Killed Hens, and Attacks

During these 79 observations, there were 141 sightings of birds of prey. Buzzards were regularly seen in groups, the maximum was a family of 5 members on farm 4, but goshawks were only observed to be solitary. Table 3 summarises the numbers of sightings per bird of prey species per farm.

During the 79 farm visits, a total of 41 inspections of free-range areas were carried out on foot, resulting in the discovery of 44 dead hens (Table 4).

During the 79 observations, a total of 10 attacks on 12 hens by birds of prey were observed, resulting in 3 hens being killed by birds of prey and 1 severely injured hen was killed by the farmer in order to prevent further suffering (Table 5).

**Figure 1:**
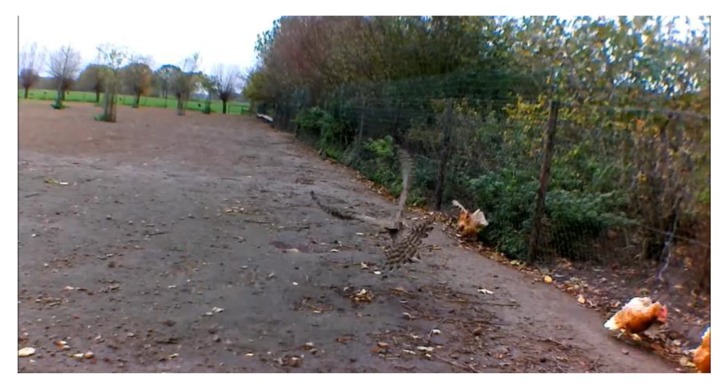
Northern goshawk (*Accipiter gentiles*) during attack 11.

After the manager of farm 9 reported that he repeatedly found carcasses of hens killed by birds of prey in the same spot, a video camera was installed that made continuous recordings. Another 6 attacks were filmed with this camera (Table 6 and Figure 1).

### 3.3. Features and Behaviour of Attacked Hens and Bystander Hens

**Figure 2:**
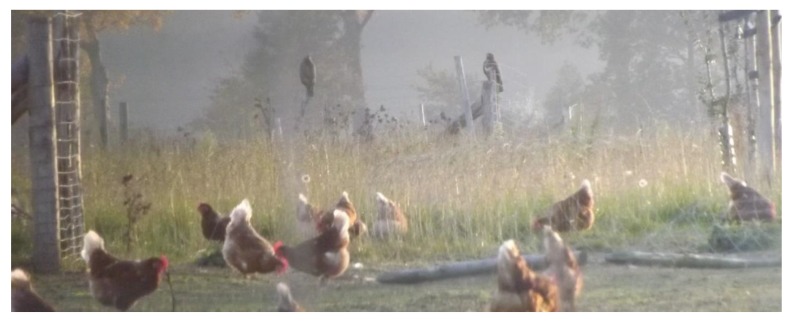
Hens on farm 8 performed normal foraging behaviour (‘walking with pecking and scratching’ [11]) while being watched by 2 common buzzards (*Buteo buteo*).

None of the hens attacked in the 16 described attacks were visibly ill, impaired, weakened or already dead. In other words: birds of prey attacked hens that were considered to be healthy (attacks 1–16). Sometimes, the initial response of hens was to drop down (attacks 4–8, 10), but most hens tried to escape or fought back (attacks 4–7, 10). Bystander hens ran away (attack 8) but were also seen trying to chase away the bird of prey (attacks 14, 15). In several instances, while the bird of prey was eating from its prey, other hens came closer and closer (attacks 11–14), sometimes to less than one metre from the scene. When a bird of prey, for example, was sitting on a pole (which was part of the fencing), hens walked underneath it and did not seem to be scared (Figure 2). Two farms (5 and 8) kept roosters and hens at a ratio of 1:30. Roosters were seen to attack and chase away birds of prey (attacks 2, 3), but they were not always in the right spot at the right moment (attack 5). Generally, bystander hens started cannibalising the killed hen as soon as the bird of prey left (attacks 1, 8, 11–14). Sometimes hens were eating from the carcass at the same time as the bird of prey (attacks 13, 14).

### 3.4. Scavengers Eating the Remains of Killed Hens

**Figure 3:**
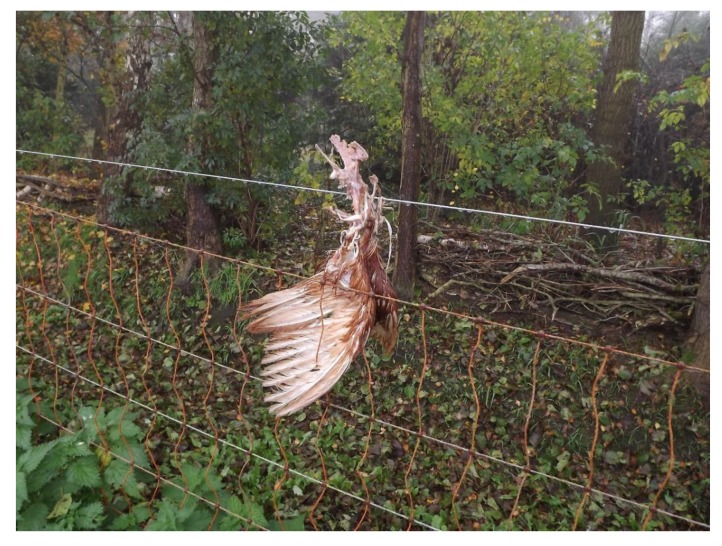
Chicken skeleton left on electric fence (farm 6).

Video recordings on farm 9 revealed that a killed hen was eaten within 2 to 3 days until a clean skeleton remained. Scavengers seen were the common buzzard (*Buteo buteo)* and hens. Some of the video recordings made in the free-range area of farm 9 showed carrion crow (*Corvus corone)*, Eurasian magpie (*Pica pica)*, red fox (*Vulpes vulpes*) and a domestic cat (*Felis silvestris catus*) to be present. In most cases, the clean skeletons were laying on the ground, but on farm 6 they were twice seen hanging from an electric fence (Figure 3). The common buzzard was mentioned in a comparable case of the remains of prey hanging on barbed wire [12].

### 3.5. Vegetation on the Free-Range Areas and Artifical Shelters

The free-range areas varied from being sparsely to largely covered with trees. Farms 3, 4 and 11 had a few small shelters on their free-range areas. Attacks took place in an open field close to a fence (attacks 1, 2, 4–6, 8, 10–16), under trees (attacks 3, 7) or close to a shelter (attack 9). In one case, an attack was observed to stop after the hen had run under a shelter (attack 9). In some of the attacks, the hen was able to escape alive (attacks 2, 3, 6, 7, 9, 10), but, because of our sample size, we cannot say whether this was related to the presence of trees or an artificial shelter.

### 3.6. Online Survey about Mortality Caused by Predation

Although a total of 61 farmers partly filled out the online survey, only 27 completed the quantitative questions about their last culled flock. Table 7 shows the contribution of several causes of death and disappearance to the mortality of hens during the laying period. The number of hens killed by predators were assumed to be the sum of the hens found dead in the free-range areas that were recognisably killed by a predator, plus the number of hens that seemed to be missing when comparing the number of hens that arrived at the slaughter house with the farm’s mortality records. It was calculated that, on average, 3.7% of the hens in organic/free-range flocks were killed by predators. The average mortality in organic/free-range flocks was 12.2%; 8.1% died because of disease and 0.3% died from other causes.

Flock size was positively correlated with the number of hens killed by predators (n = 27; R = 0.42; *p* = 0.031). However, flock size was not correlated with percentage (%) of hens killed by predators (n = 27; R = −0.24; *p* = 0.220).

### 3.7. Yield Losses due to Predation

Table 8 shows the costs and yields per organic and per free-range hen, comparing results for hens who had completed the laying period (aged 78 weeks for organic and 82 for free-range hens) with those killed halfway the laying period (49 and 51 weeks, respectively). The laying period starts at 20 weeks of age.

On 1 January 2019, 190 organic and 252 free-range farms were registered in the Netherlands [1], with 2,411,548 and 6,293,531 hens, respectively. The mean farm size was thus 12,692 for organic farms and 24,974 for free-range farms. Assuming an average 3.7% of hens killed by predators (Table 7), this results in yield losses of (0.037 × 12,692 × 12.11=) EUR 5687 for an average organic farm, and (0.037 × 24,974 × 7.26=) EUR 6709 for an average free-range farm.

## 4. Discussion

### 4.1. What Are the Bird of Prey Species that Kill Hens?

During the field observations, both common buzzards (*Buteo buteo*, hereafter referred to as ‘buzzards’) and northern goshawk (*Accipiter gentilis*, hereafter referred to as ‘goshawks’) were seen to attack and kill hens. Buzzards are known to catch small mammals, sometimes an adult rabbit, amphibians and young birds, and they also eat carrion [14]. Buzzards mostly hunt from a position high above the ground [14]. An adult hen of around 2 kg is substantial heavier than most of the prey normally caught by buzzards. However, chickens may represent easy prey, since they seem unafraid of a buzzard sitting on a fence pole or in a tree, which are some of its regular hunting positions. Goshawks are known to catch small- to medium-sized birds, but sometime also larger ones, up to the size of small geese [15]. Goshawks hunt from the air [15]. They generally do not eat carrion, although there is some evidence to the contrary [16]. An adult laying hen corresponds well to the average prey size of goshawks. In our observations, a laying hen was attacked and killed by a goshawk, but subsequently eaten by one or more buzzards, after they chased away the goshawk. Stealing or scrounging other animals’ food or prey (i.e., kleptoparasitism) is described for several animal species, including buzzards [17]. Buzzards are described as both the robber and the robbed, while goshawk is described only as the one being robbed.

We did our field observations in July to November. We cannot exclude that at other times of the year (breeding season, wintering birds from Nordic countries, variation in abundancy of alternative prey animals, possibly fewer chickens outside during rainy and windy season) birds of prey and mammalian predators might behave differently. For the answers to qualitative research questions 1 and 2, we do not expect a difference, since buzzard and goshawk, the species that attacked hens, are species that are present here year round, with additional Nordic buzzards in winter. There is anecdotical information from farmers that foxes kill more chickens during the breeding season compared to the rest of the year. Thus, depending on the season, the number of chickens killed by avian or mammalian predators may vary.

We did not see any attacks on hens in the morning. An explanation for this may be the fact that the pop-holes on many farms opened at 10 or 11 a.m. This made the morning observation period considerably shorter compared to the afternoon and evening observation periods.

### 4.2. Condition of Hens prior to Attack

If predated hens were healthy and, therefore, would likely still have been producing sellable eggs, their predation would result in a yield loss. It was not possible to check the health or productive state of hens prior to being attacked by birds of prey. However, the 16 documented attacks showed no irregularities that would indicate health problems in the hens involved. Moreover, in most of the attacks, the hens were observed to struggle in order to escape from the bird of prey, which probably would have been less the case in diseased or weakened hens. We have no reason to assume that predated hens were in poorer health or lesser productive state prior to the attack, compared to non-predated hens.

### 4.3. Impact of Predation

The impact of predation is expressed in terms of numbers of hens per flock being killed and in terms of yield losses (Euros). Since our own field data were from a few months in summer and autumn and not collected continuously (‘24/7′), they were not representative for year-round predation. Therefore, to determine the number of hens being killed per flock, the results of the online survey were used. Those results were based on culled flocks. Since the productive life of a flock of laying hens generally lasts for a minimum of one year [10], the mortality figures from the online survey covered all seasons. When necessary to interpret qualitative aspects of those mortality figures, however, we used our findings from the field observations.

#### 4.3.1. Numbers of Hens Killed

We used farm records, collected by means of an online survey, to estimate the numbers of hens being killed by predators. It was clear to the responders that the survey was about predation. In addition, although farmers without a predation problem were explicitly invited as well, it was possible that farmers who had experienced predation-related mortality, were overrepresented in the responses. All farmers that responded had experienced predation, either because they found hens killed by predators, or hens seemed to be missing after comparing the counts at the slaughterhouse with the farm mortality records. Based on our results, we cannot say what proportion of the total population of organic/free-range poultry farmers experience predation-related mortality. In a survey in the UK [3], 81% of the responding poultry and geese farmers had experienced predation. The farmers responding to that survey knew the survey was about fox predation, so an over-representation of farmers experiencing predation could not be excluded. A Dutch study on health and welfare [2] reported 40% of organic egg producers to have experienced predation-related mortality. Since this study was about health and welfare and the questions about predation constituted only a minor part of it, this Dutch study may better reflect the actual proportion of farmers experiencing predation. A French study [4], however, reported that 70% of the respondents reported predation, while when visiting the farms that did not report predation, field evidence nevertheless indicated predation on some of those farms. It remains difficult to conclude what proportion of farms experience predation.

Various comments can be made about the results of our own field observations, which are also the case for the farmers response ‘death caused by predator’ in the online survey. Concerning cadavers found in the free-range, even when showing signs of predation, it cannot always completely be excluded that the hen had died from another cause and was subsequently fed on (i.e., secondary predation). Also, there are reasons to assume that the real number of hens killed by predators is higher than the number of those observed to be killed or found dead. Observers, but also farmers, are not able to oversee the whole free-range continuously, so attacks will very likely be missed. Carcasses of killed hens seemed to disappear fast, which also makes it likely that not all of them will be detected when inspecting the free-range.

It is generally assumed that the majority of hens that seem to be missing after comparing the number of hens counted that arrived at the slaughterhouse with those in the farm mortality records are killed by predators. However, how reliable is this? First, how reliable is the number of young hens that arrived on the laying farm? This was verified by asking a representative from a rearing company that delivers young hens to organic egg production farms. He explained [18] that, from the moment of putting fertilised eggs into the hatching machine to the moment of delivering the young hens to the egg production farm, the eggs/hens will be counted several times; some of the counts are performed automatically, and they are believed to be very precise. This results in deviations in the number of delivered hens that are ‘closer to 10 than to 50′.

Second, how certain is it that farmers will find all of the hens that died on their farms? This was verified by asking a farmer who was producing both barn eggs and free-range eggs with a total of 50,000 hens. He explained [19] that, from his free-range flocks, he generally lost more than a thousand hens per flock and ‘none’ from his barn flock. Another free-range farmer, who was keeping 38,000 hens, said that, inside the hen house, he ‘rarely overlooked a dead hen’ [20]. The experiences of these 2 farmers suggest that if hens were missing, they had not disappeared from the hen house, nor were their carcasses overlooked inside the hen house.

Third, how precise is the count of the number of arrivals at the slaughterhouse? This was verified by asking the manager of a slaughterhouse processing the majority of Dutch organic hens. He explained that their automatic counting system was precise, resulting in a ‘closed count’ [21].

Fourth, assuming the missing hens disappeared from the free-range area, how likely is this to be due to predation? Other causes of disappearances from or death in the free-range areas could be disease, hens being locked out of the hen house because of pop holes closing before all hens were inside, smothering, and drowning. Concerning disease, hens who are ‘close to death’ or in pain would not be expected to be physically able or willing to leave the hen house to go outside, as was found in hens with keel fractures [22]. The risk of hens becoming locked out is generally avoided by installing automatic timers, and, if it were to happen, those dead hens would probably have been found by the farmer. Hens who died because of smothering or drowning would probably have been found by the farmer, too. Hens found dead by the farmer would be included in the farm mortality records and thus not end up as ‘missing’ after the slaughterhouse count. These considerations still suggest predation as the main cause of death in the free-range areas.

Fifth, assuming the hens had disappeared because of predation, how many of them were killed by birds of prey? Our observations of hens found dead during the inspections of the free-range areas suggested that the majority were killed by birds of prey (73%), rather than foxes (9%). A higher proportion killed by birds of prey was also found for French broiler production: 52% were killed by birds of prey and 28% were killed by ‘mammals’ [4]. A German experimental farm only described birds of prey causing predation [8] cited in [6]. Two farmers joining our study who had kept detailed records of causes of death mentioned 15% (farm 9) and 25% (farm 8) of kills having been caused by foxes and 85% or 75% by birds of prey. In contrast, in English egg production, nearly all killings were by foxes [3]. In summary, in the flocks included in our study, the most likely predators seemed to be birds of prey. This may be related to our message while recruiting farms; we were specifically looking for farms with mortality caused by birds of prey.

One of the results from our online survey was that farmers reported that 1.2% of the hens in their free-range flocks were found dead within the range area; according to the farmer, they were killed by a predator. Another 2.5% of the hens in free-range flocks seemed to be missing according to a count on their arrival at the slaughterhouse—as stated above, probably killed by a predator, in most cases a bird of prey. Taking into account the above considerations, and assuming there were no other substantial causes of death in the free-range areas, it was estimated that, on average, 3.7% (0.2 to 12.0) of the hens in free-range flocks that were included in our online survey had died because of predation, and most of them were considered to be killed by birds of prey. These numbers correspond to what other researchers found: 0.5% (up to 12%) of laying hens and geese in the United Kingdom [3], 6.3% (up to 34%) of broilers in France [4], 6.7% of laying hens in Switzerland [5] cited in [6], up to 14.2% of laying hens in Denmark [7] and 9.5 (up to 23.5%) of laying hens in Germany [8] cited in [6].

#### 4.3.2. Yield Losses

Yield losses were calculated as the difference in yield between a hen living a productive life until the day of slaughter (78 and 82 weeks for organic and free-range hens, respectively) and one living only half of its productive life. The yield losses were calculated to be EUR 12.11 per killed organic and EUR 7.26 per killed free-range hen. Furthermore, we calculated the yield losses to be roughly EUR 5700 for an average organic farm and EUR 6700 for an average free-range farm. We used average key figures, standardised prices for young hens, eggs, feed and other costs. However, the average mortality caused by predators may vary per farm; we found a mean of 3.7% of predation in a ‘population’ of 27 farms, which was likely overrepresented by farmers with predation-related mortality. The percentage of hens killed by predators in the complete population of organic/free-range farms would possibly include farms without or with less predation, as well. Calculating a mean for the complete population would then result in <3.7% of predation-related mortality. On top of that, egg price, feed costs and other costs vary from farm to farm as well.

#### 4.3.3. Starting Points for Preventive Measures

If, on average, 4% or up to 12% of the hens of a flock is being killed by predators, in our study mostly birds of prey, taking measures becomes an obvious next step. As mentioned in the introduction section, prevention of predation by birds of prey seems possible to only a limited extent. Roosters, which we observed to chase away birds of prey, were not always in the right place at the right time and could not prevent the killing of hens. Trees that could function as shelter were also used by birds of prey as a starting point for attacks. Fence poles were also used as a starting point, but they are a necessary part of the fence. In addition to attacks from starting points, attacks were also seen from open air; therefore, removing ‘physical starting points’ is not expected to be able to prevent attacks. One attack was aborted after the attacked hen ran under a shelter. To what extent such structures can be used as preventive measures is doubtful; just like the roosters, they would not always be in the right place at the right time. Although we recommend further research into preventive measures, our results do not suggest starting points for doing so for the size of farms (up to 19,000 in the ‘field study group’ and up to 46,000 in the ‘survey group’) in our study.

## 5. Conclusions

Both northern goshawks (*Accipiter gentilis*) and common buzzards (*Buteo buteo*) killed laying hens. Common buzzards were also observed to scavenge, after having chased away the northern goshawk, who had killed the particular hen. Hens that were attacked, did not show symptoms of disease or weakness prior to the attack. Moreover, in most cases, they tried to escape from their attacker. There were no reasons to assume that predated hens were in poorer health than non-predated hens. Predation was estimated to have been the cause of, on average, one third of the mortality in the organic/free-range flocks that were included in our survey; total mortality was reported to be 12.2%, of which 3.7% was estimated to be due to predators. Combining these findings with average key figures gives a rough estimate of yield losses of EUR 5700 on an organic farm (size: 12,700 hens) and EUR 6700 on a free-range farm (size: 25,000 hens) experiencing predation-related mortality.

## Figures and Tables

**Table 1:** Farms where field observations took place.

Farm	Number of Hens on the Farm (Rounded)	Genotype	Age of Hens at First and Last Observation (in weeks)	Percentage (%) of Hens Using Free-Range Area Under Favourable Conditions ^1^	Roosters	Number of 90-Min Observations	Size of Free-Range Area in Hectares	Tree Cover as % of Free-Range Surface ^2^	Vegetation and Shelters in Free-Range Area	Openness of Surrounding Landscape
1	17,000	Brown	66–73	45	No	5	6.9	<5	Grass, willow trees, maize	Half open
2	6000	Brown	57–64	90	No	4	2.4	5	Grass, adult oaks	Half open
3	12,000	Brown	52–66	25	No	10	4.8	<5	Grass, small shelters	Half open
4	19,000	Brown	45–60	50	No	10	7.5	<5	Grass, young fruit trees, small shelters	Half open
5	9000	Brown	35–49	90	Yes	9	3.4	75	Trees, grass	Open
6	15,000	Brown	55–70	45	No	8	6.0	75	Trees, shrubs, grass	Open
7	6000	Silver ^3^	66	80	No	2	2.4	<5	Grass, young trees	Half open
8	12,000	Brown	37–51	65	Yes	10	4.9	90	Young fruit trees, grass	Half open
9	13,660	Brown	26–38	75	No	9	5.4	<5	Grass, young trees, shrubs	Half open
10	11,760	Brown	32–37	50	No	6	4.7	<5	Grass, young trees	Open
11	9000	Brown	68–73	33	No	6	3.6	5	Grass, young trees, small shelters	Open

^1^ This was an estimate by the farmer, who was asked what percentage of this flock he generally sees outside under favourable conditions: before sunset with cloudy and calm weather. ^2^ Estimation based on Google Maps satellite images and photographs made in the free-range areas. ^3^ Silver hens are a reverse-cross white layer breed; they have a weight comparable to brown hens, have mostly white and a few brown feathers and lay brown eggs [13].

**Table 2:** Date and start times * (M(orning); A(fternoon); E(vening)) of 90-minutes observation periods (n = 79).

Farm	July	August	September	October	November
1	20M, 21A, 30M		7E, 9M		
2	22M, 23M		7A, 8A		
3	28A, 29M		16M, 17M	6A, 7M, 21A, 22A, 28M	3A
4	20A, 21M	5M	8M, 9M, 28M, 29A	23A ^7^, 26A	5A ^9^
5	24A, 27M		11A ^3^, 15A	1E, 5A, 19A, 29A, 30A	
6	24M, 27A		11A, 15M	1A, 2M, 29A	2M
7	22A, 23M				
8	28A, 29A		10E ^2^, 16A	6A ^5^, 8A, 15A, 22A, 27A	3A
9		6M	10A ^1^, 17A	7A, 8A ^6^, 15A, 21A, 27A, 28A	
10			28A, 29A ^4^	23A, 26A ^8^	4A, 5A ^10^
11				2A, 5A, 16A, 20A, 30M	2A

* Morning < 12:00 h; Afternoon 12:00–18:00 h; Evening > 18:00 h. ^1–10^ Attacks observed; numbers correspond with attack numbers in Table 5.

**Table 3:** Sightings of birds of prey during field observations.

Farm	Number of 90-Min Observations	Common Buzzard *Buteo buteo*	Northern Goshawk *Accipiter gentilis*	Common Kestrel *Falco tinnunculus*	Eurasian Hobby *Falco subbuteo*	White-Tailed Eagle *Haliaeetus albicilla*	Total Number of Birds of Prey
1	5	8	1	0	0	0	9
2	4	2	0	0	0	0	2
3	10	6	0	4	2	0	12
4	10	23	1	6	3	0	33
5	9	9	1	0	1	0	11
6	8	11	0	1	0	1	13
7	2	2	0	0	0	0	2
8	10	19	0	4	0	0	23
9	9	10	2	0	0	0	12
10	6	11	0	2	0	0	13
11	6	8	0	3	0	0	11
Total	79	109	5	20	6	1	141

**Table 4:** Numbers of hens found dead during inspections carried out on foot and their assumed cause of death.

Farm	Number of Inspections	Suspected Predation		Other/Unknown	Total
		Bird of Prey	Fox		
1	3	3	0	4	7
2	2	1	0	0	1
3	5	3	0	0	3
4	5	8	2	2	12
5	5	5	1	0	6
6	4	2	0	1	3
7	1	0	0	1	1
8	5	0	1	0	1
9	5	4	0	0	4
10	3	4	0	0	4
11	3	2	0	0	2
Total	41	32	4	8	44

**Table 5:** Attacks on hens by birds of prey (BOP) seen during field observations, numbered in chronological order.

Attack	Date & Time ^1^	Farm	Flock Age (weeks)	Bird of Prey Species	Condition ^2^ of Hen Prior to Attack	Predator Killed Hen	Observations
1	10 September 16:01–17:31	9	31	*Accipiter gentilis*	Healthy	Yes	Hen was attacked next to fence. Immediately after the BOP left, other hens ran to the killed hen and cannibalized it.
2	10 September 19:09–20:29	8	43	*Buteo buteo*	Healthy	No	Hen was attacked in open field, next to the fence. Immediately at the start of the attack, roosters ran to BOP and chased it away. Hen survived.
3	11 September 16:22–17:57	5	42	*Accipiter gentilis*	Healthy	No	Hens were attacked under the trees. BOP attacked 3 hens, and was chased away by roosters. Hens survived. While flying away, the BOP was also chased by *Buteo buteo*.
4	29 September 17:00–18:30	10	32	*Buteo buteo*	Healthy	Yes	BOP1 attacked hen in open field, next to fence. Hen first dropped down, but then resisted. BOP2 flew over BOP1 during attack. BOPs ate together from the hen. BOP3 flew over and disappeared.
5	6 October 17:52–19:22	8	47	*Buteo buteo*	Healthy	No	BOP attacked solitary hen in open field, next to fence, while other hens and roosters had gone inside. Hen first dropped down, but then resisted. BOP scared off by the observer and fled. Hen was euthanised by the farmer because of a severe breast wound.
6	8 October 12:55–14:52	9	35	*Accipiter gentilis*	Healthy	No	BOP attacked hen in open field, next to fence. Hen first dropped down, but then resisted, fled and got attacked again. Fled again and BOP flew into tree. Hen ran away.
7	23 October 15:30–17:00	4	58	*Accipiter gentilis*	Healthy	No	BOP attacked hen under a tree, next to fence. Hen first dropped down, but then resisted and fled, right into an electric fence. BOP flew away, flew back over hen and disappeared.
8	26 October 16:00–7:27	10	36	*Buteo buteo*	Healthy	Yes	BOP attacked hen in open field, next to fence. Hen dropped down, screamed and did not resist. Other hens ran towards hen house. BOP left hen after 15 min eating breast. Hens ran to killed hen and cannibalised it.
9	5 November 13:27–15:00	4	60	*Buteo buteo*	Healthy	No	BOP attacked hen in open field, next to artificial shelter. Hen ran under shelter. BOP disappeared.
10	5 November 15:27–17:27	10	37	*Buteo buteo*	Healthy	No	BOP attacked hen in open field. Hen first dropped down, but then resisted and after 5 min BOP disappeared. Hen ran towards hen house.

^1^ Start and end times of observations. ^2^ A hen was considered ‘healthy’ if she was displaying normal free-range behaviour (alternating standing, pecking, walking and foraging [11]) and no other behaviour or particularities were seen that would suggest the hen to be somehow impaired, diseased or weakened.

**Table 6:** Attacks on hens by birds of prey (BOP) filmed with camera, numbered in chronological order.

Attack	Date & Time	Farm	Flock Age (weeks)	Bird of Prey Killing the Hen	Condition ^1^ of Hen Prior to Attack	Observations
11	14 November 12:16–13:17	9	40	*Accipiter gentilis*	Healthy	BOP killed resisting hen in open field, close to fence. While feeding on hen, other hens were getting closer, <1 m. After 1 h, BOP left. Other hens immediately started cannibalising the killed hen.
12	21 November 12:23–13:44	9	41	*Accipiter gentilis*	Healthy	BOP killed resisting hen in open field, close to fence. After feeding on the hen for 10 min, BOP was chased away by a *Buteo buteo*. Within 1 h, 3 *Buteo buteo* were seen with the hen. More and more hens came closer to *Buteo buteo* feeding on the hen. After BOP left, other hens started cannibalising the killed hen.
13	22 November 14:37–15:28	9	41	*Accipiter gentilis*	Healthy	BOP killed resisting hen in open field, close to fence. After feeding on hen for 20 min, BOP was chased away by a *Buteo buteo*. In total, 2 *Buteo buteo* were seen with the hen. While *Buteo buteo* were feeding, other hens and magpie (*Pica pica*) also fed on the hen. BOPs left 1 h after the attack and other hens moved in and cannibalised the killed hen.
14	23 November 13:11–14:28	9	42	*Accipiter gentilis*	Healthy	BOP killed resisting hen in open field, close to fence. After feeding on the hen for 20 min, BOP was chased away by a *Buteo buteo*. While BOP was feeding, 2 hens unsuccessfully tried to chase it away, then stayed and ate blowing down feathers from the scene. More hens approached. After 1 h of feeding, BOP left and hens started cannibalising the dead hen.
15	28 November 12:53–13:23	9	42	*Accipiter gentilis*	Healthy	BOP killed resisting hen in open field, close to fence. While it was feeding from the hen, another hen tried to chase it away BOP without success. After 40 min, BOP was chased away by a *Buteo buteo*.
16	29 November 13:05–13:53	9	42	*Accipiter gentilis*	Healthy	BOP killed resisting hen in open field, close to fence. After feeding on the hen for approx. 1 h, BOP left on its own initiative.

^1^ A hen was considered ‘healthy’ if she was displaying normal free-range behaviour (alternating standing, pecking, walking and foraging [11]) and no other behaviour or particularities were seen that would suggest the hen to be somehow impaired, diseased or weakened.

**Table 7:** Causes of death of chickens in the last culled flock on organic/free-range farms.

Initial Number of Hens and Causes of Death and Disappearance	Mean Number of Hens (Minimum–Maximum)	Percentage (%) of Hens, Relative to Initial Number (Minimum–Maximum)
Initial number of hens	17,868 (200–46,000)	100
Killed by disease	1543 (3–10,371)	8.1 (1.5–41.9 ^2^)
Found dead on free-range, death caused by predator	172 (0–1400)	1.2 (0.0–5.4)
Found dead on free-range, cause of death other than by predator	29 (0–300)	0.3 (0.0–6.0)
Birds missing after comparing arrivals at slaughterhouse with farm records	406 (0–1817)	2.5 (0.0–10.0)
Mortality caused by predation ^1^	579 (5–2600)	3.7 (0.2–12.0)
Total mortality	2150 (9–12,588)	12.2 (3.3–50.8)

^1^ Mortality caused by predation is the total number of animals found dead in the free-range area after having been killed by a predator, plus those that are missing, after comparing the numbers arriving at the slaughterhouse with farm mortality records. ^2^ In one flock, mortality was extremely high due to an infection with *Pasteurella multocida*.

**Table 8:** Financial result (margin) per hen, under scenarios with and without predation, both for an organic (ORG) and a free-range (FR) production system.

Key Figure	ORG Hen Scenario NO Predation	ORG Hen Scenario WITH Predation ^1^	FR Hen Scenario NO Predation	FR Hen Scenario WITH Predation ^1^
Length of laying period (days)	406	203	434	217
Eggs/housed hen	338	169	360	180
Price/egg (€)	0.135	0.135	0.075	0.075
Feed intake (grams/hen/day)	126	126	121	121
Feed intake (kg/hen)	48.3	24.15	49.8	24.9
Feed conversion	2.33	2.33	2.25	2.25
Price/kg feed (€)	0.46	0.46	0.265	0.265
Yields (€)				
Eggs	45.63	22.82	27.00	13.50
Carcass after slaughter	0.40	0.00	0.36	0.00
Total yield	46.03	22.82	27.36	13.50
Costs (€)				
Purchase young hen	7.50	7.50	4.44	4.44
Feed	22.22	11.11	13.20	6.60
Other production costs ^2^	1.56	1.56	1.56	1.56
Interest costs ^3^	0.23	0.23	0.14	0.14
Total costs	31.51	20.40	19.34	12.74
Margin (€)	14.52	2.42	8.03	0.76
Yield reduction (€)	-	12.11	-	7.26

^1^ The predation was assumed to have taken place distributed evenly over the laying period. Thus, the same numbers of hens were assumed to be killed before and after the middle of the laying period, which meant that calculations could be done with all predation taking place ‘halfway’ through the laying period. ^2^ Other production costs include those of electricity, water, health care and hygiene, litter, monitoring, catching, and cadaver pick-up. ^3^ Interest costs were based on long-term investments related to egg production (housing, land).
